# Reconciling uncertainty of costs and outcomes with the need for access to orphan medicinal products: a comparative study of managed entry agreements across seven European countries

**DOI:** 10.1186/1750-1172-8-198

**Published:** 2013-12-24

**Authors:** Thomas Morel, Francis Arickx, Gustaf Befrits, Paolo Siviero, Caroline van der Meijden, Entela Xoxi, Steven Simoens

**Affiliations:** 1 KU Leuven Department of Pharmaceutical and Pharmacological Sciences, O&N2 bus 521, Herestraat 49, Leuven, Belgium; 2 National Institute for Health and Disability Insurance (RIZIV/INAMI), Brussels, Belgium; 3 Dental and Pharmaceutical Benefits Agency (TLV), Stockholm, Sweden; 4 Italian Medicines Agency (AIFA), Rome, Italy; 5 Health Care Insurance Board (CVZ), Diemen, The Netherlands

**Keywords:** Managed entry agreements, Orphan drugs, Orphan medicinal products, Access, Reimbursement, Risk sharing, Uncertainty, Health technology assessment, Payers, Pricing, Performance

## Abstract

**Background:**

National payers across Europe have been increasingly looking into innovative reimbursement approaches – called managed entry agreements (MEAs) – to balance the need to provide rapid access to potentially beneficial orphan medicinal products (OMPs) with the requirements to circumscribe uncertainty, obtain best value for money or to ensure affordability. This study aimed to identify, describe and classify MEAs applied to OMPs by national payers and to analyse their practice in Europe.

**Methods:**

To identify and describe MEAs, national health technology assessments and reimbursement decisions on OMPs across seven European countries were reviewed and their main characteristics extracted. To fill data gaps and validate the accuracy of the extraction, collaboration was sought from national payers. To classify MEAs, a bespoke taxonomy was implemented. Identified MEAs were analysed and compared by focusing on five key themes, namely by describing the MEAs in relation to: drug targets and therapeutic classes, geographical spread, type of MEA applied, declared rationale for setting-up of MEAs, and evolution over time.

**Results:**

42 MEAs for 26 OMPs, implemented between 2006 and 2012 and representing a variety of MEA designs, were identified. Italy was the country with the highest number of schemes (n=15), followed by the Netherlands (n=10), England and Wales (n=8), Sweden (n=5) and Belgium (n=4). No MEA was identified for France and Germany due to data unavailability. Antineoplastic agents were the primary targets of MEAs. 55% of the identified MEAs were performance-based risk-sharing arrangements; the other 45% were financial-based. Nine of these 26 OMPs were subject to MEAs in two or three different countries, resulting in 24 MEAs. 60% of identified MEAs focused on conditions whose prevalence is less than 1 per 10,000.

**Conclusions:**

This study confirmed that a variety of MEAs were increasingly used by European payers to manage aspects of uncertainty associated with the introduction of OMPs in the healthcare system, and which may be of a clinical, utilisation, or budgetary nature. It remains unclear whether differences in the use of MEAs reflect differences in how ‘uncertainty’ and ‘value’ are perceived across healthcare systems.

## Background

As a new orphan medicinal product (OMP) receives European marketing authorisation across the European Union (EU), national health technology assessment (HTA) bodies and national healthcare payers subsequently determine its value to inform or decide reimbursement. This is usually not a straightforward exercise as there is often considerable uncertainty about the ultimate real-world clinical and economic performance of that new OMP.

Often as a result of the inherent nature of rare diseases (i.e. life-threatening or chronically debilitating diseases with a prevalence of 5 out of 10,000 or less) [1], the clinical evidence package on OMPs that is submitted to national healthcare payers in the context of reimbursement applications tends to be limited relative to drugs for common diseases [2-7]. Their clinical evidence package is often associated with uncertainty at product launch due to the difficulty of recruiting a sufficient number of patients (as a result, reaching statistically significant results may be challenging), patient population is often heterogeneous, many trials of approved OMPs are only based on surrogate endpoints (e.g. time to progression, response rate or progression free survival), traditional study designs are sometimes not feasible (e.g. randomization and inclusion of control arms may be unethical), and the assessment of the observed clinical improvement may be difficult as little is usually known about the natural history of the disease.

Moreover, many OMPs are associated with relatively high treatment costs [8-14], which adds to the budgetary uncertainty dimension and/or to the financial risk to the payer in the event the treatment does not work in real life as well as anticipated. In contrast to industry-sponsored predictions stating that the rate of budget impact of OMPs is expected to grow slowly from 2010 to 2016, at which time it is expected to plateau at approximately 4.6% of total pharmaceutical market expenditure [15], a recent study across the main five EU countries suggests that OMP expenditure and utilisation are rapidly growing, particularly for some ATC groups, such as antineoplastic drugs. Findings show that in those countries both expenditure and utilisation increased in the year 2010 compared to 2009, ranging from 13 to 28% and 7 to 17% respectively [16].

Against that background of ambient uncertainty, national healthcare payers have been increasingly looking into innovative reimbursement approaches to balance the need to provide rapid access to potentially beneficial health technologies to patients with the requirements to obtain best value for money and to ensure affordability [17]. These innovative reimbursement mechanisms have been referred to by a variety of names, such as risk sharing, patient access schemes, or performance-based reimbursement agreements and have been studied extensively, with the subsequent development of a number of taxonomies [18-24]. The HTAi Policy Forum grouped these many terms under the terminology of ‘managed entry agreements’ (MEAs), defined as “*an arrangement between a [pharmaceutical] manufacturer and payer/provider that enables access to (coverage or reimbursement of) a health technology subject to specific conditions. These arrangements can use a variety of mechanisms to address uncertainty about the performance of technologies or to manage the adoption of technologies in order to maximise their effective use, or limit their budget impact*” [21]. In other words, MEAs may take a variety of forms depending on the nature of the concerns they are addressing, namely: managing budget impact; managing uncertainty relating to clinical and/or cost-effectiveness; or managing utilisation to optimise performance.

The present study, focusing on seven European countries, had three main objectives, namely to: (i) examine the processes through which MEAs are implemented by national healthcare payers, (ii) identify, describe and classify MEAs applied to OMPs by national healthcare payers, and (iii) analyse and compare identified MEAs related to OMPs within and between countries.

## Methods & taxonomy

The following European countries were included in this analysis: Belgium, England and Wales, France, Germany, Italy, the Netherlands and Sweden. These countries, spread across the northern and southern parts of the EU and characterised by different healthcare financing methods, are usually considered as priority market targets for new drug launches. They were selected to ensure representation of key healthcare markets in Europe. The inclusion of these countries in the study aimed to maximise the opportunity to capture a broad and representative range of MEAs for OMPs. Together, these countries represent approximately 60% of the rare disease population in the EU.

### Methods

The first study objective was addressed by exploring relevant national healthcare payers’ websites and repositories of national legal documents. Statutes establishing national payer bodies and documents describing national MEA processes of health technologies were searched, with particular emphasis to identify specific references to orphan technologies. Where possible, searches were conducted in local language (Dutch, English, French, German and Italian) to increase accuracy and comprehensiveness of the extraction. This approach, which focused on retrieving information from source documents, was preferred to exclusive reliance on summarised statements about MEA practices in the literature. In addition, validation of the identified process flows and policy or legal references was sought from representatives of national healthcare payers.

To identify, describe and classify MEAs applied to OMPs, three steps were implemented. Firstly, to identify relevant MEAs, HTA appraisals and reimbursement decisions from national bodies across seven European countries were reviewed as well as congresses (e.g. International Society for Pharmacoeconomics and Outcomes Research (ISPOR), Drug Information Association (DIA)) and payer bodies’ annual reports. All sources mentioning MEAs related to OMPs between 2006 and 2012 were kept. Search was conducted in multiple languages, as above. Secondly, to describe MEAs, a data extraction sheet was developed to capture the evidence needed for analysis. A data extraction protocol was followed to increase homogeneity of the extraction. MEAs were characterised in terms of the country involved, compound (i.e. name, marketing authorisation date, indication, prevalence, therapeutic subgroup (ATC levels 1 and 2)), date and outcome of the HTA appraisal or reimbursement decision, rationale for setting up a MEA, scheme details (i.e. main objectives and mechanisms, start date, endpoints used, data on possible registry). Websites of organisations such as the European Medicines Agency (EMA) or Orphanet and of clinical trials registers such as clinicaltrials.gov were also used extensively to ascertain the clinical evidence package of individual OMPs. The extraction was performed by a single researcher (TM). To complement this desk research, fill in the data gaps and validate the accuracy of the extraction, formal collaboration was sought from a selection of national payers, HTA bodies and insurers to organise structured interviews and request access to MEA-related databases. Collaboration requests were sent out to the following entities: the National Institute for Health and Disability Insurance (INAMI/RIZIV, Belgium), the Healthcare Products Pricing Committee (CEPS, France), the Institute for Quality and Efficiency in Health Care (IQWiG, Germany), the National Association of Statutory Health Insurance Funds (GKV, Germany), the Italian Medicines Agency (AIFA, Italy), the Health Care Insurance Board (CVZ, the Netherlands), the Netherlands Organisation for Health Research and Development (ZonMw, the Netherlands), the Dental and Pharmaceutical Benefits Agency (TLV, Sweden), and the National Institute for Health and Clinical Excellence (NICE, England & Wales). Thirdly, to classify MEAs related to OMPs, a literature review of existing taxonomies was performed. The taxonomy developed by the ISPOR task force on performance-based risk-sharing arrangements was selected, being the most recently developed and comprehensive in capturing the various types and subtypes of MEAs possible within a national pricing and reimbursement framework [20]. However, the chosen taxonomy was further adapted to fit the research objective and an arm related to financial-based arrangements was specified as the latter were out of scope of the ISPOR work.

Identified MEAs related to OMPs captured in the dataset were then analysed and compared by focusing on five key themes, namely by describing the MEAs in relation to: drug targets and therapeutic classes, geographical spread, type of MEA applied, declared rationale for setting-up of MEAs, and evolution over time. Data were structured in Excel format to allow for description of trends, similarities and differences according to the themes mentioned above in the total sample or stratified by country.

## Results

### Taxonomy

**Figure 1:**
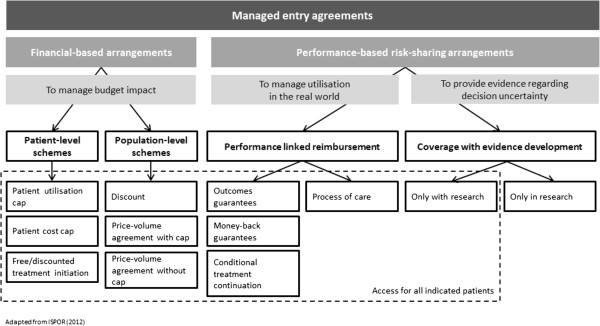
A taxonomy of managed entry agreements (MEAs).

The taxonomy used in our study distinguishes between MEAs that measure health outcomes in characterising performance (i.e. performance-based risk-sharing schemes) and those that do not consider outcomes but focus on keeping expenditure within agreed limits (i.e. financial-based arrangements) (Figure 1).

Performance-based risk-sharing schemes can be broken down into two categories: (a) ‘performance-linked reimbursement’ schemes that aim to manage utilisation and guarantee the cost-effectiveness of a new health technology in the real-world by linking performance at the individual patient level to payment or reimbursement of a new technology; and (b) ‘coverage with evidence development’, where coverage decision is conditioned upon the collection of additional population-level evidence. Within ‘performance-linked reimbursement’ , while some schemes may relate payment to ‘*process of care’* (i.e. payment is directed towards those patients that satisfy eligibility criteria for a treatment, for example as a result of a genetic test), some other schemes focus on ex-post reimbursement, measuring intermediate or clinical endpoints. The latter include: ‘*outcomes guarantees*’ (i.e. payment for responders only), ‘*money-back guarantees*’ (i.e. refund for non-responders or patients having discontinued), and ‘*conditional treatment continuation*’ (i.e. payment for continued use of the medicine for those patients reaching a pre-defined intermediate treatment milestone). ‘Coverage with evidence development’ (CED) can be further broken down into schemes that are ‘*only in research*’ (i.e. coverage is conditioned on individual participation in research), and CED schemes ‘*only with research*’ where all new patients may be treated using the new technology.

Financial-based arrangements, the second type of MEAs described in our taxonomy, aim to address concerns over the budgetary impact associated with the introduction of a new health technology. They may either adopt a patient- or a population-level perspective. The first category includes: ‘*cost capping*’ (i.e. the maximum cumulative cost of treatment per patient is specified [for a period of time] and beyond this threshold, the pharmaceutical manufacturer provides its drug at a discount or free of charge), ‘*utilisation capping’* (i.e. the total number of doses or cycles of treatment is agreed on. Any excess beyond this limit is penalised financially) and ‘*free or discounted treatment initiation*’ (i.e. therapy is free or discounted up to a specified number of doses or treatment cycles). Population-level financial-based arrangements include: ‘*discounts’* (i.e. therapy is provided by the pharmaceutical manufacturer at a reduced cost to the National Health Service for all eligible patients) and ‘*price-volume agreements’* that may come with caps or not. In price-volume agreements ‘*without cap’*, the unit price of a drug is linked to the expected volume sold (negotiated at product launch), so that it declines when volume increases. Price-volume agreements ‘*with cap’* stipulate the volume that may be sold, based on forecast sales. If the sales volume or budget is exceeded, the pharmaceutical manufacturer is penalised, usually by having the price of the drug reduced (i.e. discount) or by having to pay-back (i.e. rebate) the amount of sales above the agreed levels. There are a variety of possible payback clauses. Of note, price-volume agreements may take complex forms. For instance, a complex price-volume agreement ‘with cap’ may not be specific to a drug but set an annual sales cap for all the drugs used to treat a particular therapeutic indication, leading to rebates for any excess according to each sponsor’s market share.

### Managed entry agreements

References to national MEA processes and policy frameworks were retrieved for all seven countries in scope of analysis. A total of 42 MEAs specific to 26 OMPs, implemented between 2006 and 2012 across five European countries and representing a variety of MEA designs, were identified and reviewed. Details of these 42 MEAs are fully outlined in Additional file 1: Table S1 and described and analysed below.

Data from national health authorities on MEAs established in France and Germany were, unfortunately, unavailable. Neither the German GKV nor the French Healthcare Products Pricing Committee and Ministry of Health responded favourably to our request of research collaboration, on grounds of confidentiality. As a result, only data resulting from desk research of published data are reported here for France and Germany, which is likely to under-estimate the true incidence of MEAs related to OMPs in these countries.

### Belgium

#### MEA process

In 2010, Belgian law was amended to introduce the possibility to negotiate MEAs for Class I drugs (i.e. pharmaceutical products for which there is a claim for added therapeutic value) for which there was a negative motion for reimbursement or no motion from the Drug Reimbursement Committee within the National Institute for Health and Disability Insurance (NIHDI). Contract negotiation is motivated by an excessive reimbursement basis claimed by the applicant with regard to the considered/recognized therapeutic or social added-value or by uncertainties related to the drug budget impact [25,26]. It is up to the manufacturer to inform the Minister for Social Affairs and Health of their readiness to negotiate an MEA. Procedure involves a 120 days clock-stop period during which the text of the agreement is negotiated by a taskforce composed of the applicant, insurance bodies, representatives from the Ministries for Social Affairs and Budget, and a member of the Belgian pharmaceutical association. An MEA is only signed between NIHDI and the applicant once the two Ministers involved consent and subsequently results in the temporary enlisting of the drug, for a maximum of three years. Drug performance and budgetary impact is assessed over this period of time in view of later re-evaluation. Article 83 of the amended Royal Decree of 21 December 2001 stresses that MEAs must include some key mechanisms of action such as price-volume agreements with pay-back clauses, cross deals, or reductions of the reimbursement level. In December 2012, after two years of implementation of the new Belgian policy, 22 MEAs had been negotiated in the context of reimbursement applications by NIHDI.

#### Identified MEAs specific to orphan medicinal products

**Figure 2:**
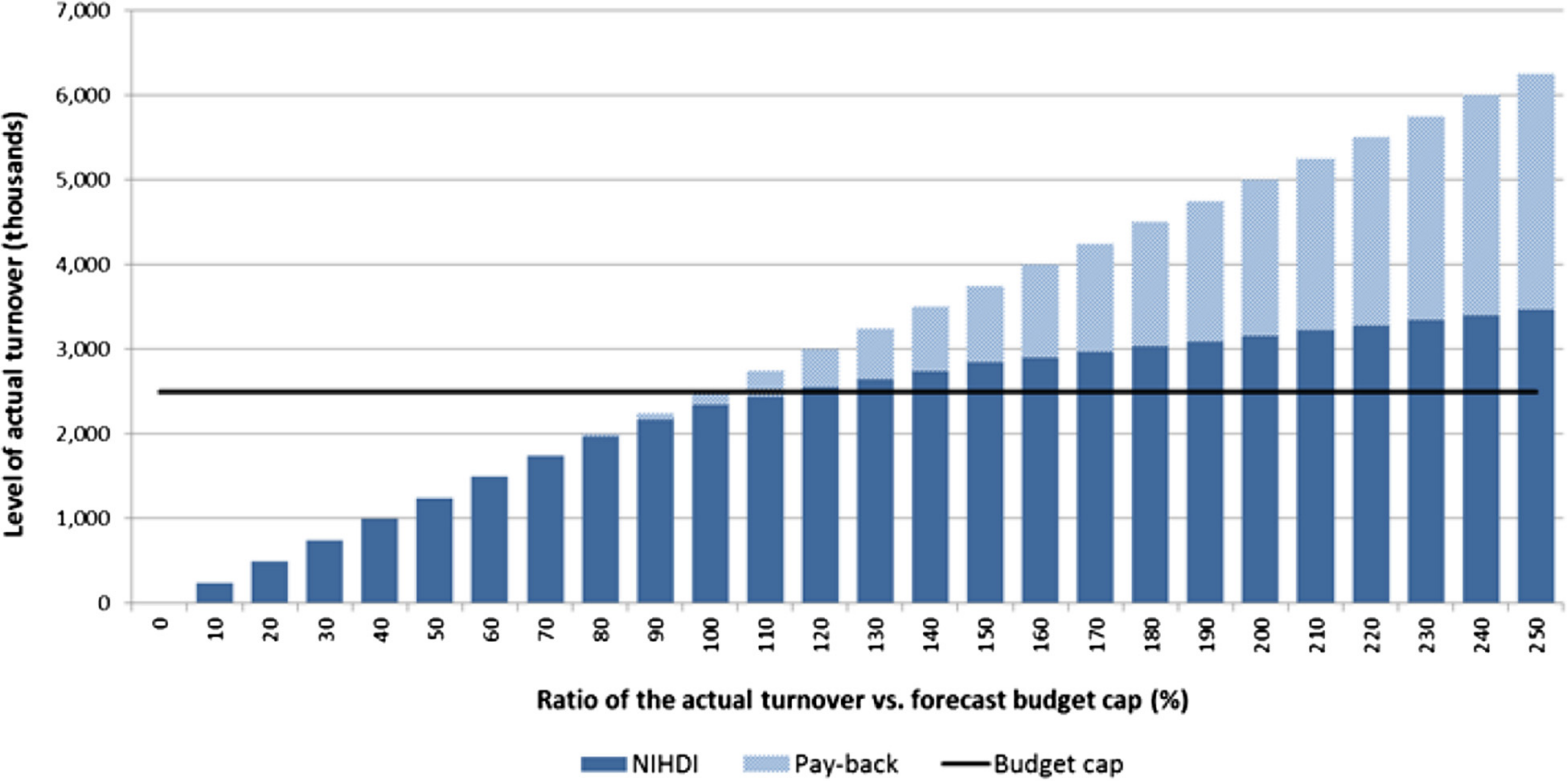
Example of a price-volume agreement ‘with cap’ established by NIHDI.

Between 2010 and 2012, four MEAs related to OMPs were negotiated with the Belgian authorities. Uncertainty related to future budget impact and high treatment cost were the main drivers for setting up these MEAs. All four contracts were financial-based arrangements and foresaw a re-evaluation of the drug reimbursement status after three years. Albeit financially-based, these schemes also included a secondary outcomes-based perspective, as they required the submission of Phase IV outcomes data at the time of re-evaluation. Two of these schemes applied an original type of price-volume agreement, which modulates incrementally the amount of pay-back as the actual turnover increases and exceeds the budget cap pre-agreed with NIHDI (Figure 2). For instance, it may be agreed between the pharmaceutical manufacturer and NIHDI that the former pays back 10% of the sales as the real turnover gets to 75% of the forecast budget, 20% once the 90% level is reached, 40% as turnover equals forecast budget, 60% as 150% of the forecast budget is reached etc. Through this pricing framework, NIHDI accepts to increase its willingness-to-pay if it appears that a larger patient population than expected can be treated and that the level of unmet medical need is equally larger. That being said, this scheme also allows NIHDI to check that the greater drug sales do not result from off-label use.

The third MEA stipulated a patient cost cap arrangement that ensured that all first-line therapies in the indication of newly diagnosed Philadelphia-chromosome-positive chronic myelogenous leukaemia were priced on a par through a refund mechanism. The fourth MEA foresees the application of a discount, coupled with a price-volume agreement.

### England & Wales

#### MEA process

In 2009, and further to prior developments for drugs indicated for multiple sclerosis and multiple myeloma [27-29], the Pharmaceutical Price Regulation Scheme (PPRS) [30] formally introduced patient access schemes (PAS) as a way to improve access to innovative treatments whose incremental cost-effectiveness ratio was initially too high to meet the requirements from the National Institute for Health and Clinical Excellence (NICE) to be recommended for use. Patient access scheme proposals are therefore made in the context of a NICE technology appraisal with the specific purpose of improving the cost-effectiveness of a drug to avoid non-recommendation by NICE. Proposals for a PAS are left to the initiative of the pharmaceutical company. PAS proposals may be introduced either at the time of initial submission to NICE or at the end of the appraisal process, once any appeals have been heard and NICE’s final guidance has been issued to the NHS. Once proposed, PAS are referred by the Department of Health to the Patient Access Schemes Liaison Unit (PASLU) within NICE who then advises whether the scheme is feasible for implementation in the NHS in England and Wales in light of a set of key principles. According to the latter, schemes must be operationally manageable, clinically robust and plausible, and without unduly complex monitoring or disproportionate administrative burden. The Department of Health and the Association of the British Pharmaceutical Industry have agreed on a bespoke typology for PAS. Importantly, PAS should be the exception rather than the rule, the PPRS insists, and particularly outcomes-based schemes. And priority is likely to be given to schemes that deliver the greatest benefits to patients, for example in enabling the NHS to address a previously unmet need.

#### Identified MEAs specific to orphan medicinal products

In December 2012, there were 33 national-level PAS in the NHS, of which eight focused on seven OMPs (i.e. 24% of total) [31]. Six of these eight PAS were directed to antineoplastic and immunomodulating agents. All of these PAS were financial-based schemes according to our taxonomy, and included a mix of patient- and population-level arrangements, namely: five discount schemes, one patient cost cap, and two patient utilisation cap schemes. The predominance of discount schemes reflects the general trend observed across the 33 national-level PAS in place – this type of scheme is the easiest to implement and was promoted through the development of an accelerated process for simple discount PAS [32]. As laid out in the PPRS, all of these PAS were set up to improve the cost-effectiveness of these new OMPs to enable recommendation by NICE. Schemes covered the totality of the target population of each indication. Of note, one performance-based scheme (and specifically a money-back guarantee) once applied to an OMP: sorafenib, for the treatment of advanced hepatocellular carcinoma. This scheme was discontinued in May 2010 after rejection by NICE [33].

### Italy

#### MEA process

Since 2006 [34], the Italian Medicines Agency (AIFA) has entered into a wide range of MEAs whenever any newly launched medicine presents some uncertainty over its clinical value/effectiveness, budget impact, or potential inappropriate use.

Management of uncertainty related to clinical benefit and effectiveness is done through the use of monitoring registries aimed at collecting data on drug prescription, administration and effectiveness. The AIFA monitoring registries are online tools that organise the exchange of data regarding the appropriate use of medicines according to their approved indications between the regulators, dispensing pharmacists, prescribing clinicians, manufacturers and Italian regions. Registries track the eligibility of patients (i.e. diagnosis and patient characteristics) and the complete treatment flow (i.e. drug dosage and administration, evaluation of treatment responders, treatment failure/end of treatment, tolerability and reported adverse events) [35]. Today, the AIFA monitoring registries cover some 80 therapeutic indications across over 60 medicines and approximately 450,000 patients.

In a move to further circumscribe possible uncertainty on clinical effectiveness (and any risk of superfluous expenditures for the NHS) AIFA made sure that these monitoring registries may also be coupled, if necessary, with performance-based reimbursement schemes, named by AIFA as ‘*Payment by results*’ and ‘*Risk sharing*’ schemes. Both types of schemes evaluate the rate of treatment ‘non-responders’. For every non-responder, the drug manufacturer is either expected to grant a discount to the cost of initial treatment cycles (risk-sharing) or to refund the full cost of therapy (payment by results). Through these agreements, AIFA and the NHS effectively shift part of the cost of evaluating new medicines in clinical practice to their manufacturers.

Furthermore, uncertainty around a drug budgetary impact may be mitigated by financial-based schemes, including ‘*price-volume agreements’* and ‘*cost-sharing agreements*’ (i.e. a discount applied to the initial cycles of therapy for all eligible patients). Lastly, to manage utilisation uncertainty, AIFA may apply ‘*Restricting notes for prescription’* whereby it restricts reimbursement to specific patient populations, or ‘*Therapeutic plans*’ that conditions reimbursement to the drug prescription by specialised health care centres.

**Figure 3:**
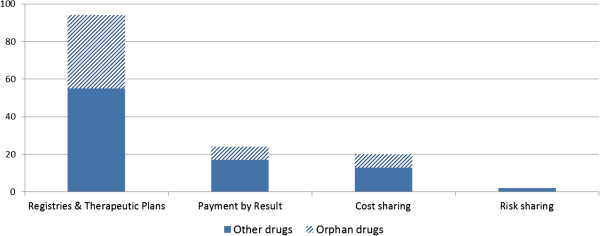
**Number of MEAs set up by AIFA, described by MEA design (according to AIFA’s terminology).** Note: A single medicine may be subject to several of these mechanisms at the same time. This graph does not show details for price-volume agreements and restricting notes as these arrangements are not applicable to orphan medicinal products. Source: AIFA, 2013.

In early 2013, across all therapeutic indications, 26 performance-based schemes existed (i.e. 24 ‘payment by results’ and 2 ‘risk sharing’), in parallel to over one hundred financial-based schemes (i.e. 20 ‘cost sharing’ and 85 ‘price volume agreements’) (Figure 3). No specific law regulates the process of decision-making on MEAs; rather, they are part of the AIFA pricing and reimbursement negotiation with the drug manufacturer and are decided on a case-by-case basis.

#### Identified MEAs specific to orphan medicinal products

In December 2012, we listed 15 MEAs applied to 10 OMPs – all antineoplastic or immunomodulating agents. Of these 15 MEAs, according to our taxonomy, eight were ‘performance-based schemes’ whereby non-response to treatment is sanctioned by a money-back guarantee; and seven other MEAs were financial-based (either ‘discounted treatment initiation’ or ‘discount’). Sorafenib, nilotinib and temsirolimus were each subject to both categories of MEAs, according to the indication involved. Within the context of ‘performance-based schemes’ , non-responders are usually identified within a timeframe of 4 to 8 weeks. Of note, in 2012, the performance-based scheme applied to dasatinib was replaced by a financial-based arrangement (i.e. discounted treatment initiation). The reported objectives of all these MEAs are to verify and control the appropriateness of the prescription, and to circumscribe the level of uncertainty around the drug. AIFA’s ‘*payment by result schemes*’ (i.e. ‘money-back guarantee’ schemes according to our taxonomy) typically suggest a higher level of uncertainty.

Evidently, all of these 10 OMPs have a monitoring registry linked to them (‘*Registro farmaci oncologici sottoposti a monitoraggio AIFA*’). For some other nine OMPs^1^, however, only the continuous monitoring of their prescription applies – they are not subject to any type of performance- or financial-based arrangement. Most of them are covered by the national monitoring registry of OMPs (‘*Registro nazionale farmaci orfani’*), which includes a broader range of pharmacotherapeutic groups, such as anti-haemorrhagic or alimentary tract and metabolism products.

### The Netherlands

#### MEA process

In 2006, the Dutch government introduced a ‘coverage with evidence development’ system, whereby drugs were initially only temporarily admitted on the expensive or orphan medicine list of the Dutch Healthcare Authority (NZa). The policy regulations on expensive and orphan medicines (‘*Beleidsregel dure geneesmiddelen*’ and ‘*Beleidsregel Weesgeneesmiddelen*’) foresaw the possibility of national subsidies to hospitals to help them finance their care services and ensure patient access to therapies. Hospitals received additional funding of 80% of the costs of these drugs (100% for OMPs). In return, applicants were required to conduct outcomes research studies to generate evidence on appropriate drug use and effectiveness in daily practice and real-world cost-effectiveness [36,37]. A yearly budget impact greater than €0.6 million per hospital was the main criterion to justify the inclusion of a new OMP into the scheme. After four years, a (re)assessment by the Dutch Healthcare Insurance Board (CVZ) had to take place to advise on the possibility to continue the national subsidies to hospitals or not.

In January 2012 the reimbursement of inpatient drugs changed, thereby putting an end to the two policy regulations on expensive and orphan medicines. Medicines in hospital settings with annual costs per patient of €10,000 or more are now reimbursed via ‘add-on’ funding, whereby physicians of university hospitals are entitled to claim ‘add-on’ drug expenses to treat specific rare diseases. Conditional and temporary entry with the requirement to conduct outcomes research, however, has been maintained and even strengthened [38]. It now applies across all inpatient drugs that have a budget impact of €2.5 million or more and for which the estimates for (cost) effectiveness are assessed by CVZ as being surrounded by an unacceptable level of uncertainty.

#### Identified MEAs specific to orphan medicinal products

In 2012, some 23 medicines (across 32 therapeutic indications) and 11 OMPs were included in the Dutch policy regulation on expensive medicines and policy regulation on orphan medicines respectively [39]. Details of ten out of these eleven coverage with evidence development (CED) schemes are available in Additional file 1: Table S1 (NB: the CED scheme applied to canakinumab was not analysed as it is no longer an OMP). All ten MEAs can be categorised as CED schemes ‘only with research’ according to our taxonomy, meaning that positive coverage decision is conditional upon the collection of additional evidence across all identified patients – usually over a four-year period – to support continuation or withdrawal of coverage. Half of them were targeted towards enzyme replacement therapies; the other half involved antineoplastic and immunomodulating drugs. In addition to the criterion related to the €0.6 million budgetary threshold, the need of additional long-term effectiveness data usually drives the decision for setting up the outcomes research schemes. As illustrated in Additional file 1: Table S1, the requirements in terms of study design and outcomes are usually extensive and prescriptive, encompassing clinical, quality of life, resource use, and cost data. Sometimes, data from international registries or studies (e.g. the International Hunter Outcome Survey) is used in combination to Dutch data. Cost-effectiveness analysis is usually derived from these outcomes research studies to inform on value for money.

At the end of 2012, three OMPs had gone through the (re-)assessment and appraisal process at CVZ, namely alglucosidase alfa for Pompe disease and agalsidase alfa and agalsidase beta for Fabry disease. Study results for non-classical Pompe disease indicated that on overall patient group level efficacy was limited and that responder thresholds had not been reached [40,41]. Overall, the assessment committee determined that, on average, these OMPs had limited therapeutic added-value for the majority of patients. Moreover, limited therapeutic value was contrasted with high costs, leading to ICERs of €3 million per QALY for treatment with agalsidase alfa or beta and €15 million per QALY for treatment with alglucosidase alfa for non-classical Pompe disease. Both ICERs were determined in comparison to standard-of-care treatments. Once the assessment reports were established, the appraisal process was initiated. In June 2012, CVZ confidentially sent out draft appraisal reports to key stakeholders. In this concept advice CVZ proposed that the proof for effectiveness was too limited to justify the high drug costs. These draft reports leaked to the press, which led to a public outcry across the Netherlands and the rare disease community [42-44]. In the appraisal committee meeting interested parties were invited to express their viewpoints on the reports, and these were taken into account in the advice to the Dutch Ministry of Health. Subsequent debate has led to questioning both the ethical dimension of decisions to withdraw reimbursement in areas of high unmet needs and the relevance of outcomes research studies in patients affected by rare conditions that are limited to the boundaries of a single country such as the Netherlands. Paucity of data resulting from the low number of patients may make it difficult to reach indisputable scientific conclusions [45]. However, the reassessment was not limited to outcome research as the results from outcome research were assessed alongside relevant published (randomized) clinical trials. The assessment conclusions, therefore, were based on all relevant evidence regarding effectiveness, cost-effectiveness, and appropriate use available. At time of writing, continued coverage of these OMPs was being negotiated between the Dutch government and the respective pharmaceutical manufacturers, whereby price reductions were sought after [46].

### Sweden

#### MEA process

In October 2002 the Swedish ‘Act on Pharmaceutical Benefits’ [47] came into effect and the Dental and Pharmaceutical Benefits Agency (TLV) subsequently started its role as Sweden’s independent agency responsible for reimbursement and pricing decisions of new medicines^2^. Until recently^3^, TLV’s remit was limited to prescription drugs. The three primary criteria that TLV applies in making decisions are: equity, need and solidarity and cost-effectiveness. There is no specific policy for OMPs in reimbursement decisions, which means that any manufacturer of a new OMP must comply with the same requirement as any other medicine. However, in addition to a more lenient stance on the level of submitted evidence for OMPs compared to that for non-orphans and the acceptance of higher cost per quality-adjusted life years ratios in some instances [48,49], TLV also agreed on setting up a number of CED schemes whereby temporary reimbursement is granted in return for the collection of additional real-life data by the manufacturer to reduce the level of uncertainty surrounding the new medicine.

#### Identified MEAs specific to orphan medicinal products

From June 2003 to April 2010, TLV received requests for reimbursement for 30 OMPs. It awarded reimbursement to 29, six of which were reimbursed with limitations. Only one drug was denied reimbursement [49,50]. Since 2006, five CED schemes were set up by TLV to manage some form of uncertainty surrounding the evidence package of new OMPs. Only one is on-going (i.e. everolimus). The main uncertainties that led to the development of these schemes were (i) the need of real-life data (e.g. adherence to treatment, number of patients treated, drug dosage) and (ii) the need to validate the assumptions used in the ex-ante cost-effectiveness model submitted to TLV. According to our taxonomy these are CED schemes ‘only with research’ where all new patients must be treated using the new technology while new data are generated. Timeframe for generating new data ranged between two and three years. TLV was usually not prescriptive as to the outcomes data that it wished to see re-submitted but usually offered informal protocol assistance to manufacturers to develop the data generation plan. In theory, failure to comply with these data requirements may result in delisting. To our knowledge, this has not happened.

### France

#### MEA process

Based on article L.162-16-4 of the French Social Security Code, the retail price of a drug is set by means of a negotiated agreement between the pharmaceutical company selling the drug and the French Healthcare Products Pricing Committee (Comité économique des produits de santé, CEPS). The primary considerations for price setting are any additional medical benefit which the drug provides, the prices of other drugs providing the same treatment, and the forecast or recorded sales volumes of the drug. For innovative medicines given a strong incremental medical improvement rating (i.e. with ASMR I to III), these agreements guarantee that the price set will be no lower than the lowest price in force in the four main comparable EU markets [51]. In return, drug listing may be conditioned to volume clauses, whereby the pharmaceutical company undertakes to compensate by means of clawback payments for any additional costs to national health insurance in the event that its actual sales volume exceeds the forecast volume level mentioned in the price submission application. Similarly, price reductions and Phase IV studies may be required.

#### Identified MEAs specific to orphan medicinal products

In 2008, as a steady increase in the sales of OMPs was observed, CEPS “*questioned the value of continuing to provide support and special benefits for medicines that make a high turnover when their profitability on the market is at least as firmly guaranteed as that of most non-orphan medicines*” [52]. As a result, CEPS decided to propose ad hoc agreements to the pharmaceutical companies concerned whereby they would undertake to supply their medicine to all patients who might benefit from it without restriction, whilst paying back to national health insurance ‘the whole’ turnover they make above an agreed fixed ceiling [53]. This mechanism was implemented on two occasions in 2008: the first involved galsulfase (treatment for mucopolysaccharide type VI disease); the second related to eculizumab (for paroxysmal nocturnal haemoglobinuria). According to our taxonomy, these schemes could be defined as ‘price-volume agreements with cap’. Two years later, a legal amendment was introduced to the contractual framework between CEPS and the pharmaceutical industry, with a specific focus on access to OMPs. Article 10bis of the amended framework stated: “*to ensure that patients continue to have access to orphan medicines under conditions that are acceptable to the pharmaceutical companies and the national health insurance alike, […] the committee [CEPS] may request a company selling an orphan medicine costing more than €50K per patient per year […] to undertake to supply the medicine to all patients eligible for the treatment without restriction in return for setting a price in keeping with standard international prices, up to a set turnover threshold”*[54]*.* With this provision, the ‘capping’ approach per patient per year thus gained a legal dimension. Therefore, in France, MEAs are traditionally price-volume contracts.

### Germany

#### MEA process

Since 2003, Germany’s Statutory Health Insurance (SHI) providers have had the option to negotiate individual rebate or discount contracts with pharmaceutical companies. This option to allow SHI funds, instead of the Federal Joint Committee (G-BA), to negotiate contracts directly was confirmed with the ‘Act to Reinforce Competition between the German Statutory Health Insurance’ (GKV-WSG) of 2007. As a result, MEAs in Germany have taken place at the regional or individual sick fund level, not at the national level. Both financial-based and performance-guarantee types of MEAs were reportedly implemented [18].

With the Act on the Reform of the Market for Medicinal Products (AMNOG) coming into force in January 2011, the procedure of ‘early benefit assessment’ became mandatory to obtain reimbursement for new drugs in Germany. In case of additional benefit, price negotiations follow between the pharmaceutical manufacturer and the Federal Association of Statutory Health Insurance Funds. The additional medical benefit has been regarded as proven for OMPs, as a result of the marketing authorization by EMA.

#### Identified MEAs specific to orphan medicinal products

Experience on reimbursement and pricing negotiations under AMNOG is limited. In July 2012, pirfenidon became the second medicine (and first OMP) to be priced within Germany’s new reimbursement system. Negotiation outcome was an 11% rebate (on top of a 16% mandatory rebate) [55]. The contract has a duration of two years.

### Comparative analysis

If we consider those countries where MEA-related data were accessible (i.e. Belgium, England and Wales, Italy, the Netherlands and Sweden), then a total of 42 MEAs applied to 26 OMPs were available for analysis. Italy was the country with the highest number of schemes (n=15), followed by the Netherlands (n=10), England and Wales (n=8), Sweden (n=5) and Belgium (n=4).

Across this sample of MEAs, performance-based risk-sharing arrangements (n=23, 55% of total) were slightly more prevalent than financial-based schemes (n=19) (Table 1). Performance-based risk-sharing arrangements were relatively more common in Italy, the Netherlands and Sweden; financial-based schemes were mainly encountered in Belgium, England and Wales, and Italy. Overall, if we consider the adapted taxonomy of MEAs used in this research, we observe that, except for ‘price-volume agreement without cap’, every other possible type of financial mechanism was used across these five countries. In contrast, out of the six possible performance-based MEAs, ‘CED with research’ and ‘money-back guarantees’ were the only two utilised forms observed.

**Table 1:** Overview of MEAs identified across five European countries, described by country and design

**Types of MEAs**	**Countries**					**Number of MEAs**
	**B**	**E**	**I**	**NL**	**S**	
**Performance-based arrangements**						**23**
Performance-linked reimbursement schemes						8
*Money-back guarantees*			x			*8*
Coverage with evidence development (CED)						15
*CED ‘only with research’*				x	x	*15*
**Financial-based arrangements**						**19**
Patient-level financial schemes						10
*Discounted treatment initiation*			x			*6*
*Patient utilisation cap*		x				*2*
*Patient cost cap*	x	x				*2*
Population-level financial schemes						9
*Discount*	x	x	x			*7*
*Price-volume agreement with budget cap*	x					*2*
**Grand total**	4	8	15	10	5	**42**

B: Belgium; E: England & Wales; I: Italy; NL: Netherlands; S: Sweden.

As described in the previous sections, the rationale for setting up a MEA differs across countries. If we revert to our taxonomy, CED schemes aim ‘to provide evidence regarding decision uncertainty’, with Sweden and the Netherlands requiring real-life data to generate evidence on appropriate drug use or validation of a cost-effectiveness model. Financial-based arrangements focus on ‘managing budget impact’, with the implementation of patient cost caps, discounts, or discounted treatment initiation primarily adopted by England and Wales, Italy, or Belgium. MEAs aimed at the ‘management of utilisation in the real-world’ (by assessing drug performance through intermediate or clinical endpoints) were found only in Italy.

Antineoplastic agents were by far the primary targets of MEAs (n=23), followed by immunostimulants (n=6) and enzyme-replacement therapies (n=5). The 42 MEAs reviewed applied to 26 OMPs, which suggests that some OMPs were subject to several MEAs across countries. Nine of these 26 OMPs were subject to MEAs in two or three different countries, resulting in 24 MEAs (i.e. 57% of the total number of MEAs mapped) across seven different types of MEAs. MEAs limited to a single country were applied to the remaining 17 OMPs.

**Figure 4:**
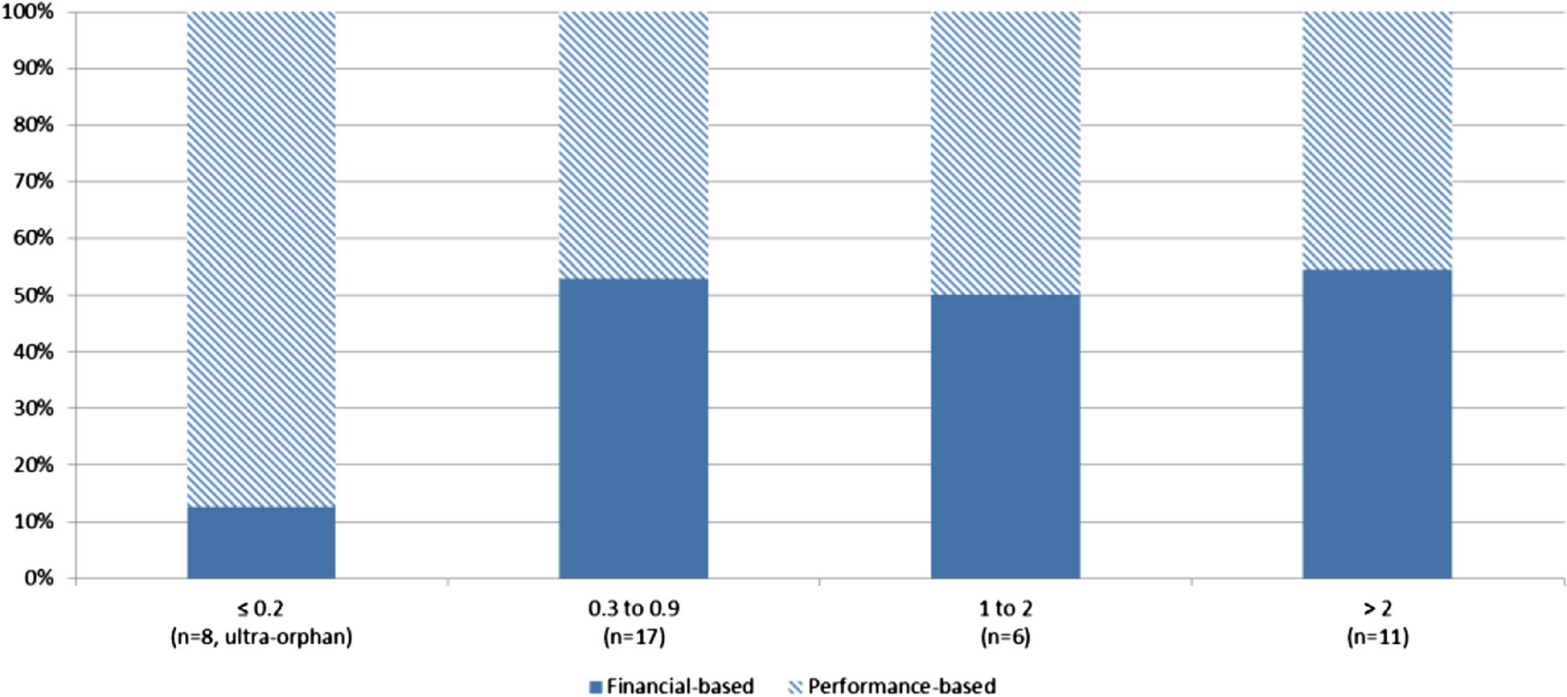
Proportion of MEAs, described by design and prevalence of the target indication (per 10,000).

25 MEAs focused on conditions whose prevalence is less than 1 per 10,000 (NB: a third of these applied to ultra-orphan medicinal products that target conditions with a prevalence equal or smaller than 0.2 per 10,000^4^) (Figure 4). The other 17 MEAs applied to OMPs aimed at conditions with prevalence equal or superior to 1 individual per 10,000. Almost all ultra-orphan medicinal products captured in our sample were subject to a performance-based MEA – an intuitive finding since in the case of ultra-orphan medicinal products clinical performance is of greater concern to healthcare payers than financial burden (owing to the very low number of patients). A 50% split between financial- and performance-based MEAs is observed for the other OMPs.

**Figure 5:**
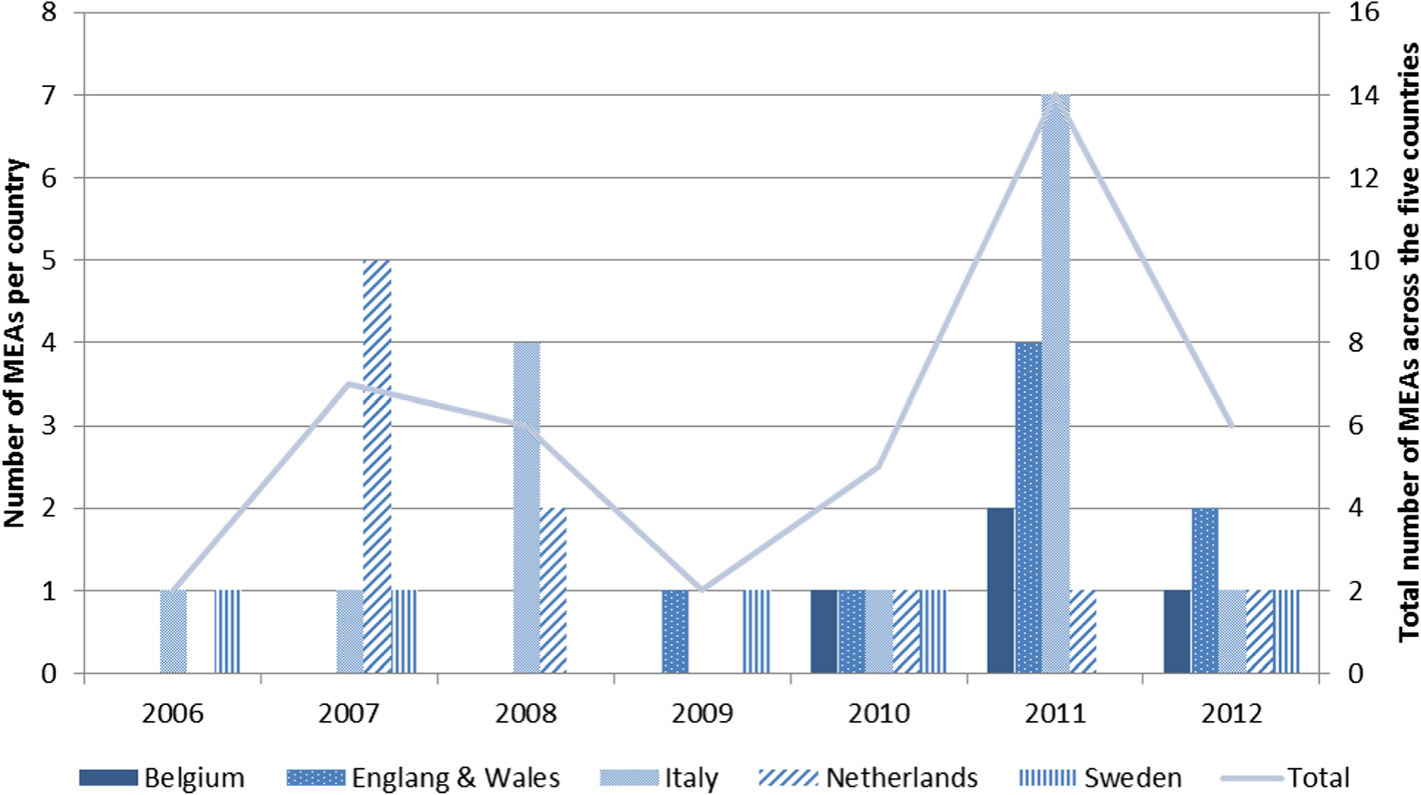
Evolution of the number of MEAs applied to orphan medicinal products over time, described by country.

The number of MEAs across the five countries has steadily increased since 2006, with a relative drop in 2012 (mainly observed in England and Italy) (Figure 5). Over this period of time, their number grew as national laws or practices establishing the use of MEAs were being adopted or implemented. Countries differed in terms of the time at which MEA frameworks were implemented, however, by 2010 all five countries had a MEA framework in place.

## Discussion

To the best of our knowledge, this study is the first published study to review and analyse the practice of managed entry agreements (MEAs) applied to orphan medicinal products (OMPs) across key European countries – a research priority area according to the World Health Organisation [56]. This research has benefited from a close collaboration with key national healthcare payers, ensuring the comprehensiveness and validity of the dataset and results. This study offers, through the use of a bespoke taxonomy of MEAs, a granular overview of the practice of MEAs, including their focus, design and geographical and temporal spread.

This study confirmed that a variety of MEAs are increasingly used by national healthcare payers to manage aspects of uncertainty associated with the introduction of OMPs in the healthcare system, and which may be of a clinical, utilisation, or budgetary nature. MEAs are, de facto, tactical tools that allow for flexibility: flexibility for national payers to earn more certainty on the value brought by an OMP and/or to reduce the bill, flexibility for pharmaceutical manufacturers to make sure that their drugs are allowed to enter the markets and reach the patients in need. By offering a framework for reaching compromises between payers and industry, MEAs make it possible to avoid the dry and dichotomous decision between ‘reimburse’ and ‘not reimburse’, and allow faster patient access to innovative therapies.

The concepts of ‘uncertainty’ and ‘value’, when applied to health technology and a fortiori to OMPs, are prone to interpretation and influenced by clinical, economic, political and socio-economic aspects. This study observed that only nine out of the 26 OMPs were enrolled in MEAs in more than two countries. Our research does suggest that the use of MEAs varied across healthcare systems, although it is not clear whether this reflects differences from when MEA frameworks were introduced and/or whether it also reflects differences in how uncertainty and value are perceived across healthcare systems. In addition, it remains to be seen what the true incidence of MEAs for OMPs is in France and Germany. This may suggest a need for a common European base for assessing the value and uncertainty level of OMPs in the EU and for sharing that information more widely across European and national regulatory and pricing and reimbursement bodies. On this very topic, it may be worth mentioning here the work at European level under the European Commission’s Working Group on the Mechanism of Coordinated Access to Orphan Medicinal Products (MoCA) to agree on an ‘European Transparent Value Framework’ to improve informed appraisal and decision-making on pricing and reimbursement across EU Member States [57-59]. This process may be paving the way to value-based pricing for orphan medicinal products. The MoCA initiative adds to the conceptual framework of the Clinical Added Value of Orphan Medicinal Products (CAVOMP) which sets the scene for improved information flows between the EMA and national HTA and payer bodies [60]. Both policy initiatives, that complement the on-going work by EUnetHTA, may re-orientate the practice of managed entry agreements in the future.

National frameworks for pharmaceutical pricing and reimbursement are currently in flux and aim to define a new footing as they are faced with increasing budgetary constraints and limited health gain with most new drugs [61]. The decline in the number of MEAs observed in 2012 in Figure 5 may result from this situation. It is yet too early to suggest whether financial-based or performance-based MEAs will become more prevalent in future, as our analysis found a somewhat similar proportion of both types of MEAs across the countries under study. While Poland [62] and Ireland [63], for example, recently started implementing performance-based risk sharing arrangements, other countries, such as the Netherlands, are considering moving away from CED schemes to price-volume agreements and other forms of performance-based pricing schemes [64,65]. Of note, the system of ‘add-on’ funding that was recently started in the Netherlands and that de facto allows for the monitoring of the volume of OMPs used in hospital actually makes it easier to implement price-volume agreements in future.

There are several limitations of this study worth noting. First, while retrieval and analysis of MEAs was possible for Belgium, England and Wales, Italy, the Netherlands and Sweden, the sensitive nature of MEAs prevented any insights into the French and German MEA frameworks. This hinders the generalisability of our comparative analysis. Second, it proved difficult to review and standardise the rationales for setting up MEAs, again as a result of data sensitivity and confidentiality. Third, the selection of the taxonomy used in this paper and its subsequent adaptation result from a literature review that was not done systematically, although we believe that all key publications on the topic of MEAs had then been retrieved and analysed. Fourth, no member from the pharmaceutical industry was consulted or involved in the course of this research. This study therefore specifically reflects the views from academia and payers. Lastly, this analysis focused on MEAs applied to orphan medicinal products: it does not compare trends in MEAs between orphan- and non-orphan medicinal products. Parallels with the recent review of MEAs in Europe – commissioned by European Commissioner Tajani in the context of his Corporate Social Responsibility initiative for pharmaceuticals [66] – proved difficult as a result of differences in geographical scope and methodology^5^.

While bearing in mind the findings of this study, a number of recommendations aimed at good practices in MEAs may be outlined. First, MEAs should have the primary goal of enabling the effective provision of an innovative and promising medicine to patients under specific conditions and within an agreed timeframe. They should, nonetheless, only apply to a restricted number of medicines. Second, they should remain voluntary contracts and should not be imposed unilaterally. As such, they should be flexible tools that may complement or replace the need for cost-containment measures. Third, the rationale, objectives and scope of MEAs should be explicit and transparent, as should its methods for review and criteria for ending the agreement. Fourth, when coverage with evidence development is opted for, then the option for cross-border patient registries – that pool patient data across several countries – should be fully investigated in order to optimise data generation while avoiding duplication of efforts. Lastly, clinical development plans should aim at addressing or attempting to address some of the main areas of uncertainty (e.g. drug performance over longer follow-up periods, clinical relevance of endpoints, quality of life, and resource use) as early as possible.

### Ethics approval

Approval from an ethical committee was not required for this descriptive research.

## Endnotes

^1^Nelarabine, idusulfase, sapropterin, mifamurtide, romiplostim, nitisinone, eltrompobag, eculizumab, and thalidomide.

^2^TLV is also responsible for medical devices, dental procedures, and for reassessing medicines that were launched on the Swedish market before 2002.

^3^In 2011, the TLV was entrusted to assess all drugs irrespective of whether they are prescribed or used in inpatient care only.

^4^The wide range of conditions that fall within the definition of ‘orphan diseases’ has led to the emergence of an informal subcategory – called ultra-orphan diseases – to describe extremely rare conditions. The term has no formal legal definition but treatments for these very rare – ultra-orphan diseases – have become known as “ultra-orphan medicinal products”. An ultra-rare disease is generally considered one that affects fewer than 20 patients per one million of population.

^5^The report by Ferrario et Kanavos (2013) confirmed that a variety of MEAs are used by national healthcare payers to tackle uncertainty. Convergence with our study findings includes: MEAs primarily aimed at antineoplastic and immune-modulating agents; performance-based schemes were slightly more prevalent. The Ferrario report did not investigate trends in the number of MEAs over time.

## Competing interests

The authors declare that they have no competing interests. However, the majority of authors are employed by health authorities. No author received financial assistance with writing this paper.

## Authors’ contributions

TM and SS designed this study and managed data collection and analysis. TM drafted the first proposed manuscript. The other authors were involved in data collection and complemented the initial drafts with referential data and expert input. All authors read and approved the final manuscript.

## Supplementary Material

Additional file 1: Table S1 Review of identified managed entry agreements (MEAs) applied to orphan medicinal products, described by country. Click here for file
