# Supplementary material for: Reconciling uncertainty of costs and outcomes with the need for access to orphan medicinal products: a comparative study of managed entry agreements across seven European countries
Source: Orphanet J Rare Dis. 2013 Dec 24;8:198. doi: 10.1186/1750-1172-8-198 (PMC3882782; doi:10.1186/1750-1172-8-198)
Supplement: Additional file 1: Table S1 — Review of identified managed entry agreements (MEAs) applied to orphan medicinal products, described by country. [file 1750-1172-8-198-S1.docx]

Additional file 1: Table S1. Review of identified managed entry agreements (MEAs) applied to orphan medicinal products, described by country

| **Compound**  **(brand name)**  **[manufacturer]** | **Therapeutic indication(s)** | **ATC category**  **(1^st^ level)** | **ATC category**  **(2^nd^ level)** | **Date of 1^st^ EMA marketing authorisation** | **EU prevalence**  **(per 10,000)** | **Date & outcome of HTA appraisal or reimbursement decision** | **Reason for setting up a MEA** | **Status of the MEA** | **MEA details** | **Patient registry details or additional outcomes research plan** | **Scheme type** |
| --- | --- | --- | --- | --- | --- | --- | --- | --- | --- | --- | --- |
| **BELGIUM** | | | | | | | | | | | |
| **Dasatinib (Sprycel®)**  **[Bristol-Myers Squibb]** | Treatment of newly diagnosed Ph+ chronic myelogenous leukaemia in the chronic phase | Antineoplastic and immunomodulating agents | Antineoplastic agents | Nov. 2006 | 0.9 | December 2011, reimbursed | High daily cost per patient compared with the other first-line therapy available | Scheme initiated in Dec. 2011 (due end: Nov. 2014) | Manufacturer needs to reimburse the difference in treatment cost with the other first-line therapy available | Manufacturer needs to collect and report by Nov. 2014 the following outcomes data: number of patients treated, dosage, additions/switches of therapy, number of discontinued treatments. | **Patient cost cap** |
| **Icatibant (Firazyr®)**  **[Shire]** | Symptomatic treatment of acute attacks of hereditary angioedema in adults (with C1-esterase-inhibitor deficiency). | Blood and blood forming organs | Other haematological agents | July 2008 | 3.0 | November 2010, reimbursed | Budget impact uncertainty | Scheme initiated in Nov. 2010 (due end: Nov. 2013) | A budget cap and a series of incremental compensation levels per block of turnover were pre-agreed between INAMI/RIZIV and the manufacturer. Every year, the latter is to pay back a proportion of the actual turnover, which increases as turnover increases and exceeds pre-agreed budget cap. | Manufacturer needs to collect and report by Nov. 2013 the following outcomes data: number of patients treated, weekly acute attacks registered, weekly used syringes by attack, type of treated angio-oedeme, treatment prevention and additional treatment of attack | **Price-volume agreement, with cap** |
| **Pirfenidone**  **(Esbriet®)**  **[InterMune UK]** | Treatment of light to moderate idiopathic pulmonary fibrosis in adults | Antineoplastic and immunomodulating agents | Immunosuppressants | Feb. 2011 | 3.0 | December 2012, reimbursed | Little evidence available | Scheme initiated in Dec. 2012 initiated  (due end: Dec. 2015) | A fixed discount was agreed in addition to a budget cap. Any excess to the pre-defined budget limit leads to a 100% pay-back. | Manufacturer needs to collect and report by Dec. 2015 the following data: data on identification of patients to benefit from treatment and responders; actual clinical benefit in real-life. | **Discount, coupled with price-volume agreement with cap** |
| **Temsirolimus (Torisel®)**  **[Pfizer]** | Treatment of adult patients with relapsed and /or refractory mantle-cell lymphoma | Antineoplastic and immunomodulating agents | Antineoplastic agents | Nov. 2007 | 0.4 | September 2011, reimbursed | Limited therapeutic added value; No information on quality of life; High treatment cost and high uncertainty on budget impact (i.e. size of target population and posology) | Scheme initiated in Sept. 2011 (due end: Aug. 2014) | A budget cap and a series of incremental compensation levels per block of turnover were pre-agreed between INAMI/RIZIV and the manufacturer. Every year, the latter is to pay back a proportion of the actual turnover, which increases as turnover increases and exceeds pre-agreed budget cap. | Outcomes data from 15 Belgian patients included in EMA Phase IV study n°B1771007 will need to be reported to INAMI/RIZIV. | **Price-volume agreement, with cap** |
| **ENGLAND & WALES** | | | | | | | | | | | |
| **Amifampridine (Firdapse®) [BioMarin]** | Treatment of Lambert-Eaton myasthenic syndrome | Nervous system | Other nervous system drugs | Dec. 2009 | 0.1 | Not reviewed by NICE yet | n.d. | Scheme initiated in Nov. 2011 | The treatment cost for each patient is capped at a maximum price in a 12 month period (equivalent to 14 packs). Once the annual cap of 14 packs has been reached, subsequent packs are supplied free of charge by the manufacturer until the 12 month period is complete. | n/a | **Patient cost cap** |
| **Azacitidine (Vidaza®)**  **[Celgene]** | Treatment of myelodysplastic syndromes, chronic myelomonocytic leukaemia and acute myeloid leukaemia | Antineoplastic and immunomodulating agents | Antineoplastic agents | Dec. 2008 | 2.3 | Reviewed by NICE (TA218) in Mar. 2011, recommended for use | Azacitidine considered as cost-effective when provided with a discount in a revised patient access scheme.  *[NB: no major concerns over clinical evidence reported by NICE]* | Scheme initiated in Jan. 2011 | Manufacturer is to make azacitidine available at a reduced cost to the NHS. The terms of this cost reduction agreement are not disclosed. | n/a | **Discount** |
| **Lenalidomide (Revlimid®)**  **[Celgene]** | Treatment of relapsed multiple myeloma | Antineoplastic and immunomodulating agents | Immunostimulants | June 2007 | 1.3 | Reviewed by NICE (TA171) in June 2009, restricted use | Average cost of treatment with lenalidomide to the NHS per person over a modelled lifetime (median overall survival approximately 2.7 years) decreased from £59,800 to £51,800 with the patient access scheme. (§3.21)  *[NB: higher ICER threshold was accepted as lenalidomide is accepted as a life-extending, end-of-life treatment]* | Scheme initiated in Jan. 2009 | NHS funds 26 cycles of treatment (about 2 years). Any treatment required beyond that threshold is entirely covered by the manufacturer, free of charge. | n/a | **Patient utilisation cap** |
| **Mifamurtide (Mepact®)**  **[Takeda]** | Treatment of osteosarcoma | Antineoplastic and immunomodulating agents | Immunostimulants | March 2009 | 0.5 | Reviewed by NICE (TA235) in Oct. 2011, recommended for use | Patient access scheme allowed for an improved ICER (§3.24)  *[NB: Appraisal mentions some uncertainty about the estimates of disease-free survival and*  *overall survival, making interpretation of data more difficult]* | n.d. | Manufacturer is to make mifamurtide available at a reduced cost to the NHS. The terms of this cost reduction agreement are not disclosed. | n/a | **Discount** |
| **Nilotinib (Tasigna®)**  **[Novartis]** | Treatment of chronic myeloid leukaemia (imatinib intolerant/resistant) | Antineoplastic and immunomodulating agents | Antineoplastic agents | Nov. 2007 | 0.24 | Reviewed by NICE (TA241) in Jan. 2012, recommended for use | Nilotinib considered as cost-effective with a patient access scheme in place (§4.3.23) | n.d. | Manufacturer is to make nilotinib available at a reduced cost to the NHS. The terms of this cost reduction agreement are not disclosed. | n/a | **Discount** |
| **Nilotinib (Tasigna®)**  **[Novartis]** | First line treatment of chronic myeloid leukemia | Antineoplastic and immunomodulating agents | Antineoplastic agents | Nov. 2007 | 0.24 | Reviewed by NICE (TA251) in Apr. 2012, recommended for use | “*Dasatinib was not cost effective and nilotinib was on the border of cost effectiveness when the patient access scheme was applied*”(§4.3.13) | n.d. | Manufacturer is to make nilotinib available at a reduced cost to the NHS. The terms of this cost reduction agreement are not disclosed. | n/a | **Discount** |
| **Romiplostim (Nplate®)**  **[Amgen]** | Treatment of chronic immune or idiopathic thrombocytopenic purpura (ITP) | Blood and blood forming organs | Antihaemorrhagics | Feb. 2009 | 1.0 | Reviewed by NICE (TA221) in Apr. 2011, restricted use | Romiplostim shown to be cost-effective only for patients with severe refractory ITP. Submitted RCTs did not provide clear evidence about relative effectiveness. | n.d. | Manufacturer is to make romiplostim available at a reduced cost to the NHS. The terms of this cost reduction agreement are not disclosed. | n/a | **Discount** |
| **Trabectedin (Yondelis®) [PharmaMar]** | Treatment of advanced soft tissue sarcoma | Antineoplastic and immunomodulating agents | Antineoplastic agents | Sept. 2007 | 0.6 | Reviewed by NICE (TA185) in Feb.2010, recommended for use | Patient access scheme allowed for an improved ICER | Scheme initiated in Feb. 2010 | The manufacturer is to supply the sixth and any further treatment cycle of trabectedin to the NHS free of charge. | n/a | **Patient utilisation cap** |
| **FRANCE** | | | | | | | | | | | |
| Inventory of recent MEAs in France was not possible as a result of poor transparency. Please refer to full text. | | | | | | | | | | | |
| **GERMANY** | | | | | | | | | | | |
| Inventory of recent MEAs in Germany was not possible as these arrangements are set up by private insurance companies. Please refer to full text. | | | | | | | | | | | |
| **ITALY** | | | | | | | | | | | |
| **Azacitidine (Vidaza®)**  **[Celgene]** | Treatment of myelodysplastic syndromes, chronic myelomonocytic leukaemia and acute myeloid leukaemia | Antineoplastic and immunomodulating agents | Antineoplastic agents | Dec. 2008 | 2.3 | Nov. 2010, reimbursed | To verify the appropriateness and control the correctness of the prescription. Drug associated with some uncertainty. | Scheme initiated in Sept. 2011 | For each registered patient being eligible to therapy, the manufacturer reimburses 11% of the cost of the drug used for the first three therapy cycles. First follow-up at the sixth therapy cycle.  *[AIFA MEA taxonomy: cost sharing]* | Monitoring registry was established (Registro farmaci oncologici) | **Discounted treatment initiation** |
| **Brentuximab vedotin**  **(Adcetris®)**  **[Takeda]** | Treatment of adult patients with relapsed or refractory CD30+ Hodgkin lymphoma | Antineoplastic and immunomodulating agents | Antineoplastic agents | Oct. 2012 | 0.2 | Oct. 2012, reimbursed  (Law 948/96) | To verify the appropriateness and control the correctness of the prescription. Drug associated with a high level of uncertainty. | Not yet activated | For registered patients not responding to therapy or having discontinued therapy (within four treatment cycles), the manufacturer needs to reimburse the cost of the first month of treatment.  [AIFA MEA taxonomy: payment by result] | Monitoring registry was established (Registro farmaci oncologici) | **Money-back guarantee** |
| **Dasatinib (Sprycel®)**  **[Bristol-Myers Squibb]** | Treatment of newly diagnosed (Ph+) chronic myelogenous leukaemia (CML) in the chronic phase; ALL (Ph+) CML, CML in lymphoid blast phase CML | Antineoplastic and immunomodulating agents | Antineoplastic agents | Nov. 2006 | CML: 0.9  ALL: 0.7 | Nov. 2011, reimbursed | To verify the appropriateness and control the correctness of the prescription. Drug associated with some uncertainty. | Scheme initiated in Jan. 2012 | For each registered patient being eligible to therapy, the manufacturer reimburses 50% of the cost of the drug used for the first three months/therapy cycles.  *[AIFA MEA taxonomy: cost sharing]* | Monitoring registry was established (Registro farmaci oncologici) | **Discounted treatment initiation** |
| **Dasatinib (Sprycel®)**  **[Bristol-Myers Squibb]** | Treatment of ALL (Ph+) CML, CML in lymphoid blast phase CML | Antineoplastic and immunomodulating agents | Antineoplastic agents | Nov. 2006 | CML: 0.9  ALL: 0.71 | May 2007, reimbursed | To verify the appropriateness and control the correctness of the prescription. Drug associated with a high level of uncertainty. | Scheme active between June 2010 and Dec. 2011 | For registered patients not responding to therapy or having discontinued therapy (assessed after four weeks), the manufacturer needs to reimburse the cost of the first month of treatment.  *[AIFA MEA taxonomy: payment by result]* | Monitoring registry was established (Registro farmaci oncologici) | **Money-back guarantee** |
| **Lenalidomide (Revlimid®) [Celgene]** | Treatment of multiple myeloma | Antineoplastic and immunomodulating agents | Immunostimulants | June 2007 | 1.3 | Feb. 2008, reimbursed | To verify the appropriateness and control the correctness of the prescription. Drug associated with some uncertainty. | Scheme initiated in Mar. 2008 | For each registered patient, manufacturer reimburses 50% of the price of the drug used for the first two treatment cycles. First follow-up within eight weeks.  [AIFA MEA taxonomy: cost sharing] | Monitoring registry was established (Registro farmaci oncologici) | **Discounted treatment initiation** |
| **Nilotinib (Tasigna®) [Novartis]** | Treatment of chronic phase and accelerated phase Ph+ CML with resistance or intolerance to prior therapy including imatinib | Antineoplastic and immunomodulating agents | Antineoplastic agents | Nov. 2007 | 0.24 | Aug. 2008, reimbursed | To verify the appropriateness and control the correctness of the prescription. Drug associated with a high level of uncertainty. | Scheme initiated in Nov. 2009 | For registered patients not responding to therapy or having discontinued therapy (assessed after four weeks), the manufacturer needs to reimburse the cost of the first month of treatment.  *[AIFA MEA taxonomy: payment by result]* | Monitoring registry was established (Registro farmaci oncologici) | **Money-back guarantee** |
| **Nilotinib (Tasigna®) [Novartis]** | Treatment of newly diagnosed Ph+ CML in the chronic phase | Antineoplastic and immunomodulating agents | Antineoplastic agents | Sept. 2010 | 0.24 | Nov. 2011, reimbursed | To verify the appropriateness and control the correctness of the prescription. Drug associated with some uncertainty. | Scheme initiated in June 2012 | For each registered patient being eligible to therapy, the manufacturer reimburses 50% of the cost of the drug used for the first three months / three therapy cycles.  *[AIFA MEA taxonomy: cost sharing]* | Monitoring registry was established (Registro farmaci oncologici) | **Discounted treatment initiation** |
| **Ofatumumab (Arzerra®)**  **[GlaxoSmithKline]** | Treatment of chronic lymphocytic leukaemia in patients refractory to fludarabine and alemtuzumab | Antineoplastic and immunomodulating agents | Antineoplastic agents | Apr. 2010 | 3.5 | May 2011, reimbursed | To verify the appropriateness and control the correctness of the prescription. Drug associated with some uncertainty. | Scheme initiated in Sept. 2011 | For each registered patient being eligible to therapy, the manufacturer reimburses 50% of the whole treatment cost (corresponding to 12 infusions).  *[AIFA MEA taxonomy: cost sharing]* | Monitoring registry was established (Registro farmaci oncologici) | **Discount** |
| **Plerixafor (Mozobil®)**  **[Genzyme]** | Indicated in combination with G-CSF to enhance mobilisation of haematopoietic stem cells | Antineoplastic and immunomodulating agents | Immunostimulants | July 2009 | 0.6 | Nov. 2011, reimbursed | To verify the appropriateness and control the correctness of the prescription. Drug associated with a high level of uncertainty. | Scheme initiated in Dec. 2011 | Manufacturer needs to reimburse all drug costs in case of treatment failure. Criteria used for assessing treatment response: patients achieving ≥ 2 x 10^6^ CD34+ cells/kg. Effectiveness is only evaluated in patients who have completed at least two doses on consecutive days.  *[AIFA MEA taxonomy: payment by result]* | Monitoring registry was established (Registro farmaci oncologici) | **Money-back guarantee** |
| **Sorafenib (Nexavar®)**  **[Bayer]** | Treatment of advanced renal cell carcinoma | Antineoplastic and immunomodulating agents | Antineoplastic agents | July 2006 | 3.0 | Nov. 2006, reimbursed | To verify the appropriateness and control the correctness of the prescription. Drug associated with some uncertainty. | Scheme initiated in Dec. 2006 | For each registered patient being eligible to therapy, the manufacturer is to reimburse 50% of the cost of the drug used for the first three months / three therapy cycles.  *[AIFA MEA taxonomy: cost sharing]* | Monitoring registry was established (Registro farmaci oncologici) | **Discounted treatment initiation** |
| **Sorafenib (Nexavar®)**  **[Bayer]** | Treatment of hepatocellular carcinoma | Antineoplastic and immunomodulating agents | Antineoplastic agents | July 2006 | 1.0 | June 2008, reimbursed | To verify the appropriateness and control the correctness of the prescription. Drug associated with a high level of uncertainty. | Scheme initiated in Aug. 2011 | For registered patients not responding to therapy or having discontinued therapy (assessed after two months of treatment), the manufacturer needs to reimburse the full cost of the drug used for the first two months of treatment.  *[AIFA MEA taxonomy: payment by result]* | Monitoring registry was established (Registro farmaci oncologici) | **Money-back guarantee** |
| **Temsirolimus (Torisel®)**  **[Pfizer]** | Treatment of advanced renal cell carcinoma | Antineoplastic and immunomodulating agents | Antineoplastic agents | Nov. 2007 | 3.5 | Sept. 2008, reimbursed | To verify the appropriateness and control the correctness of the prescription. Drug associated with a high level of uncertainty. | Scheme initiated in Apr. 2011 | For registered patients not responding to therapy or having discontinued therapy (assessed by or after eight weeks of treatment), the manufacturer needs to reimburse the full cost of the drug used for the first two months of treatment.  *[AIFA MEA taxonomy: payment by result]* | Monitoring registry was established (Registro farmaci oncologici) | **Money-back guarantee** |
| **Temsirolimus (Torisel®)**  **[Pfizer]** | Treatment of adult patients with relapsed and/or refractory mantle-cell lymphoma | Antineoplastic and immunomodulating agents | Antineoplastic agents | Nov. 2007 | 0.4 | Aug. 2011, reimbursed | To verify the appropriateness and control the correctness of the prescription. Drug associated with some uncertainty. | Scheme initiated in June 2012 | For each registered patient being eligible to therapy the manufacturer needs to reimburse the cost of the first six vials.  *[AIFA MEA taxonomy: cost sharing]* | Monitoring registry was established (Registro farmaci oncologici) | **Discounted treatment initiation** |
| **Trabectedin (Yondelis®) [PharmaMar]** | Treatment of advanced soft tissue sarcoma | Antineoplastic and immunomodulating agents | Antineoplastic agents | Sept. 2007 | 0.6 | Jan. 2009, reimbursed | To verify the appropriateness and control the correctness of the prescription. Drug associated with a high level of uncertainty. | Scheme initiated in Oct. 2011 | For patients not responding to therapy (assessed by or after eight weeks of treatment), the manufacturer needs to reimburse the full cost of the drug used for the first two therapy cycles (each cycle has a duration of three weeks).  *[AIFA MEA taxonomy: payment by result]* | Monitoring registry was established (Registro farmaci oncologici) | **Money-back guarantee** |
| **Trabectedin (Yondelis®) [PharmaMar]** | Treatment of ovarian neoplasms | Antineoplastic and immunomodulating agents | Antineoplastic agents | Sept. 2007 | 2.4 | Mar. 2011, reimbursed | To verify the appropriateness and control the correctness of the prescription. Drug associated with a high level of uncertainty. | Scheme initiated in Oct. 2011 | For patients not responding to therapy (by completion of third treatment cycle), the manufacturer needs to reimburse the full cost of the drug used for the first three therapy cycles (each cycle has a duration of three weeks).  *[AIFA MEA taxonomy: payment by result]* | Monitoring registry was established (Registro farmaci oncologici) | **Money-back guarantee** |
| **THE NETHERLANDS** | | | | | | | | | | | |
| **Alglucosidase alfa**  **(Myozyme®)**  **[Genzyme]** | Treatment of Pompe disease | Alimentary tract and metabolism | Other alimentary tract and metabolism products | Mar. 2006 | 0.13 | Recommended for use (conditional), July 2006  Final CVZ advice after re-submission review, Nov. 2012 | Need of real-world data (cost-effectiveness; drug use in daily clinical practice) | Scheme active between Feb. 2007 and Feb. 2011 | 1. Observational, non-randomized, open label, phase IV study designed to evaluate, in a real-world setting, usage and cost patterns and outcomes associated with Myozyme treatment. 2. Prospective study in patients with the non-classical form of Pompe disease (n=145). 3. Retrospective and prospective survey in patients with the non-classical form of Pompe disease (n=271, of which 99 Dutch patients). 4. Prospective randomized clinical study Late-Onset Treatment study (LOTS) (n= 90 patients), 78 weeks, main clinical endpoints: 6MWT and FVC. | n/a | **Coverage with evidence development, only with research** |
| **Agalsidase alfa (Replagal®)**  **[Shire]** | Treatment of Fabry disease | Alimentary tract and metabolism | Other alimentary tract and metabolism products | Aug. 2001 | 0.03 | Recommended for use (conditional), May 2007  Final CVZ advice after re-submission review, Nov. 2012 | Need of real-world data (cost-effectiveness; drug use in daily clinical practice) | Scheme active between June 2007 and June 2011 | Outcomes research was meant to provide insights into the effectiveness, costs and utilities of enzyme replacement therapy (ERT) for patients with Fabry disease. Two (partly overlapping) cohorts were studied (n= 142). Registry from the Amsterdam centre of expertise was used.   1. Prospective study in patients with ERT treatment follow-up>6 months (n=75). 2. From the 75 patients, comparative analysis of 58 symptomatic patients having started ERT treatment before onset of complications with data from natural history cohort (retrospective study, n=42 patients). | n/a | **Coverage with evidence development, only with research** |
| **Agalsidase beta (Fabrazyme®)**  **[Genzyme]** | Treatment of Fabry disease | Alimentary tract and metabolism | Other alimentary tract and metabolism products | Aug. 2001 | 0.03 | Recommended for use (conditional), May 2007  Final CVZ advice after re-submission review, Nov. 2012 |  | Scheme active between June 2007 and June 2011 |  | n/a | **Coverage with evidence development, only with research** |
| **Clofarabine**  **(Evoltra®)**  **[Genzyme]** | Treatment of acute lymphoblastic leukaemia (ALL) | Antineoplastic and immunomodulating agents | Antineoplastic agents | May 2006 | 0.4 | Recommended for use (conditional), Sept. 2007  *[Note: Re-evaluation of evidence after four years is limited to drugs with large budget impact. Since Evoltra’s budget impact proved moderate, it will not be re-evaluated].* | Need of real-world data (cost-effectiveness; drug use in daily clinical practice) | Scheme active between Jan. 2008 and Jan. 2012 | 1. Prospective study to include all ALL patients (age <21 y) treated with chlofarabine in the Netherlands. 2. Retrospective study of Dutch patients in the BIOV111 study (Open-Label Study of Clofarabine in Paediatric Patients with Refractory / Relapsed ALL) data to be used as control group. | n/a | **Coverage with evidence development, only with research** |
| **Eculizumab**  **(Soliris®)**  **[Alexion]** | Treatment of paroxysmal nocturnal haemoglobinuria | Antineoplastic and immunomodulating agents | Immunostimulants | June 2007 | 0.1 | Recommended for use (conditional),  Aug. 2008 | Need of real-world data (cost-effectiveness; drug use in daily clinical practice) | Scheme active between June 2008 and June 2012 | Prospective and retrospective, observational register study of eculizumab treatment and best supportive care in PNH patients. | n/a | **Coverage with evidence development, only with research** |
| **Galsulfase (Naglazyme®)**  **[Biomarin]** | Treatment of Mucopolysaccharidosis VI | Alimentary tract and metabolism | Other alimentary tract and metabolism products | Jan. 2006 | 0.02 | Recommended for use (conditional), May 2007 | Need of real-world data (cost-effectiveness; drug use in daily clinical practice) | Scheme active between June 2007 and June 2011 | 1. Prospective MPS register study. 2. Retrospectively, the international clinical surveillance program was to provide additional data and long-term follow up of clinical effects. | n/a | **Coverage with evidence development, only with research** |
| **Idursulfase (Elaprase®)**  **[Shire]** | Treatment of Hunter syndrome (Mucopolysaccharidosis II) | Alimentary tract and metabolism | Other alimentary tract and metabolism products | Jan. 2007 | 0.02 | Recommended for use (conditional), May 2007 | Need of real-world data (cost-effectiveness; drug use in daily clinical practice) | Scheme active between June 2007 and June 2011 | 1. Prospective MPS register study. 2. International Hunter Outcome Survey. | n/a | **Coverage with evidence development, only with research** |
| **Mifamurtide**  **(Mepact®)**  **[Takeda/Nycomed]** | Treatment of high-grade resectable non-metastatic osteosarcoma after macroscopically complete surgical  Resection | Antineoplastic and immunomodulating agents | Immunostimulants | Mar. 2009 | 0.5 | Not yet reviewed by CHF/CVZ | (not available) | Scheme initiated in May 2012 | (not available) | n/a | **Coverage with evidence development, only with research** |
| **Ofatumumab (Arzerra®)**  **[GlaxoSmithKline]** | Treatment of chronic lymphocytic leukemia | Antineoplastic and immunomodulating agents | Antineoplastic agents | Apr. 2010 | 3.5 | Recommended for use (conditional), June 2011 | Need of real-world data (cost-effectiveness; drug use in daily clinical practice) | Scheme initiated in Sept 2011.  End due in Sept. 2015 | 1. An observational, non-randomized open-label study designed to evaluate, in a real-world setting, usage and cost patterns and outcomes associated with Arzerra treatment. The data will be primarily collected through the PHAROS database both prospectively and retrospectively. 2. The Hx-CD20-406 study 3. Other effectiveness studies that are being carried out to satisfy EMA requirements | n/a | **Coverage with evidence development, only with research** |
| **Trabectedin (Yondelis®) [PharmaMar]** | Treatment of advanced soft tissue sarcoma | Antineoplastic and immunomodulating agents | Antineoplastic agents | Sept. 2007 | 0.6 | June 2008, first submission (negative); May 2010, 2^nd^ submission: recommended for use (conditional) | Need of real-world data (cost-effectiveness; drug use in daily clinical practice) | Scheme initiated in July 2010.  End due in July 2014 | Observational, non-randomized, open label, multicenter, register study designed to evaluate, in a real-world setting, usage patterns and outcomes associated with trabectedin treatment. Primary objective is to collect clinical data on symptomatic and best response, including tumor control rate, survival, TTP and PFS.  To make a comparison to patients receiving best supportive care, the EORTC STBSG will be assessed and 20 patients will be evaluated retrospectively. | n/a | **Coverage with evidence development, only with research** |
| **SWEDEN** | | | | | | | | | | | |
| **Deferasirox (Exjade®) [Novartis]** | Treatment of chronic iron overload | Various | All other therapeutic products | Aug. 2006 | 2.7 | Dec. 2006, reimbursed | Uncertainty on whether oral formulation would result in better adherence than infusion | Scheme active between Dec. 2006 and Dec. 2009 | The manufacturer was to provide data on utility gains and improved adherence compared to deferoxamine. | n/a | **Coverage with evidence development, only with research** |
| **Everolimus (Votubia®) [Novartis]** | Subependymal giant-cell astrocytoma | Antineoplastic and immunomodulating agents | Antineoplastic agents | Sept. 2011 | 1.0 | Apr. 2012, reimbursed | Lack of evidence (no phase III data) and uncertainty on projected cost offsets due to reduced need for surgery | Scheme initiated in April 2012. End due in June 2015 | The manufacturer is to submit to TLV a health economic analysis that includes data from clinical studies C2485 and M2301 and from clinical practice. | n/a | **Coverage with evidence development, only with research** |
| **Icatibant**  **(Firazyr®)**  **[Shire]** | Acute attacks of hereditary angioedema (HAE) | Blood and blood forming organs | Other haematological agents | July 2008 | 3.0 | Mar. 2010, reimbursed | Uncertainty on drug usage (i.e. drug may be used in a prophylactic fashion rather than for treating HAE attacks) | Scheme active between Mar. 2010 and Aug. 2011 | The manufacturer was to provide TLV with data on drug use in clinical practice. Data should show: (a) types of HAE attacks treated with icatibant, (b) number of patients treated, and (c) drug usage per patient | n/a | **Coverage with evidence development, only with research** |
| **Mecasermin (Increlex®)**  **[Ipsen Pharma]** | Severe primary insulin-like-growth-factor-1 deficiency | Systemic hormonal preparations | Pituitary and hypothalamic hormones and analogues | Aug. 2007 | 2.0 | Dec. 2007, reimbursed | Uncertainty on the assumptions of the cost-effectiveness model | Scheme active between Dec.  2007 and Mar. 2010 | The manufacturer was to provide outcomes and quality of life data (i.e. to validate model assumptions). | n/a | **Coverage with evidence development, only with research** |
| **Stiripentol (Diacomit®) {Biocodex]** | Adjunctive therapy for refractory generalized tonic-clonic seizures in patients with severe myoclonic epilepsy | Nervous system | Antiepileptics | Jan. 2007 | 0.4 | May 2009, reimbursed | Uncertainty on treatment effect | Scheme active between May 2009 and Dec. 2011 | The manufacturer was requested to provide to TLV by the end of 2011 with the results of a post-marketing study that was requested by EMA, and to have these results translated in an adapted cost-effectiveness model. | n/a | **Coverage with evidence development, only with research** |
| n.d. not disclosed | | | | | | | | | | | |
| n/a not applicable | | | | | | | | | | | |
